# Bioactive Restorative Materials Applied over Coronal Dentine—A Bibliometric and Critical Review

**DOI:** 10.3390/bioengineering10060731

**Published:** 2023-06-19

**Authors:** Paula Maciel Pires, Thamirys da Costa Rosa, Mariana Batista Ribeiro-Lages, Maysa Lannes Duarte, Lucianne Cople Maia, Aline de Almeida Neves, Salvatore Sauro

**Affiliations:** 1 Department of Pediatric Dentistry and Orthodontics, Universidade Federal do Rio de Janeiro, Rio de Janeiro 21941-853, Brazil; paulinha_pmp@hotmail.com (P.M.P.); thamirys_rosa@hotmail.com (T.d.C.R.); mbatistaribeiro@gmail.com (M.B.R.-L.); maysalannes@id.uff.br (M.L.D.); rorefa@terra.com.br (L.C.M.); aline.dealmeidaneves@gmail.com (A.d.A.N.); 2 Dental Biomaterials & Minimally Invasive Dentistry, Departamento de Odontologia, CEU Cardenal Herrera University, 46115 Valencia, Spain

**Keywords:** dental material, dentin, bibliometrics, dentin, dentistry, smart materials, tooth demineralization

## Abstract

The objective of the research was to examine the scientific literature concerning restorative materials with bioactive properties for the purpose of covering dentin. Searches were performed in various databases including MEDLINE, Scopus, Web of Science, Cochrane Library, Lilacs/BBO, and Embase. Inclusion criteria involved studies that utilized the terms “dentin” and “bioactive”, along with “ion-releasing”, “smart materials”, “biomimetic materials” and “smart replacement for dentin”. The information extracted included the title, authors, publication year, journal and the country of affiliation of the corresponding author. The studies were categorized based on their study design, type of material, substrate, analytical method, and bioactivity. A total of 7161 records were recovered and 159 were included for data extraction. Most of the publications were in vitro studies (*n* = 149), testing different types of materials in sound dentine (*n* = 115). Most studies were published in *Dental Materials* (*n* = 29), and an increase in publications could be observed after the year 2000. Most of the articles were from the USA (*n* = 34), followed by Brazil (*n* = 28). Interfacial analysis was the most investigated (*n* = 105), followed by bond strength (*n* = 86). Bioactivity potential was demonstrated for most tested materials (*n* = 148). This review presents insights into the current trends of bioactive materials development, clearly showing a severe lack of clinical studies.

## 1. Introduction

Many types of restorative materials are available for filling dental cavities, and in fact, restorations are still the most frequently performed dental treatments [1]. Dental caries reduces the mineral content (e.g., carbonate and magnesium) and changes the crystal orientation of the inorganic phase of dentine [2]. The mineral depletion exposes collagen fibers, leading to the rapid destruction of the organic part of the dentinal tissue, with a consequent risk of pulp exposure [3]. Although modern dental materials for the reconstruction of teeth have been developed, such as adhesion-based composites, glass polyalkenoate cements and ceramics [1], unfortunately, there are currently no clinically approved restorative materials that promote and regulate a specific biological response to produce reactionary dentine, to substantially increase the hardness of soft carious dentine or to induce the remineralization of collagen-depleted dentine [4].

In fact, it is desirable that restorative materials exhibit bioactivity, which would allegedly improve the mechanical properties and bond strength of the tooth–material interface. These are dependent on the dissolution behavior of the released ions and interactions triggering toughening mechanisms of crack deflection and dentine bridging microstructure morphology [5,6]. The bioactivity of dental materials relates to their potential to induce specific and intentionally desired mineral attachment to the dentine substrate, whether carious or not [7].

The ultimate goal for the restoration of tooth structure depends on the use of durable, adhesive and aesthetically acceptable materials [8]. It would be highly beneficial to develop a new generation of bioactive and therapeutic dental materials with functionalities to suppress demineralization and promote remineralization [9]. It is likely that scientific developments will improve bioactive materials formulations by incorporating specific compounds, e.g., bioactive glasses, that can rapidly release specific ions to improve the longevity of dental restorations, and/or heal dental hard tissues [10,11]. Other compounds, such as antimicrobial agents, calcium compounds, polymers and peptides, are also under investigation [7].

A significant volume of research studies featuring bioactive materials is available in research databases. Nevertheless, the absence of consensus on this subject underscores the importance of conducting a bibliometric analysis to map the existing scientific evidence regarding the bioactivity of such materials. This analysis can help identify global research trends and identify knowledge gaps in the field. Therefore, the objective of this bibliometric review is to identify the predominant research topics and groups, while critically examining the progression of scientific literature across different material types. The ultimate goal is to provide guidance for future research in this area, facilitating informed decision making and promoting the development of effective and safe dental materials.

## 2. Materials and Methods

### 2.1. Information Sources and Search Strategy

An advanced search was performed until October 2021 in several databases: MEDLINE (PubMed), Scopus (Elsevier), Web of Science Core Collection (Web of Science), The Cochrane Library (Wiley), LILACS (Virtual Health Library), BBO (Virtual Health Library) and Embase (Elsevier). This bibliographic search was carried out using MeSH terms, synonyms and entry terms for PubMed, which were then adjusted to adhere to the syntax rules of each respective database (Table 1). No filters, limits or language restrictions were applied during this stage. Alerts were created in the databases to indicate new searches, including articles published after October 2021.

### 2.2. Eligibility Criteria

Laboratory investigations, both randomized and non-randomized clinical trials, case series and case reports were considered for inclusion in this review. Specifically, studies focusing on biomodified restorative materials used in class I cavities or on flat coronary dentin were included. To ensure relevance to the review topic, these studies needed to employ specific standardized search terms such as “dentin”, “bioactive”, “ion releasing”, “smart materials”, “biomimetic materials” or “smart dentin replacement”. The use of these terms, along with their variations, in the title, abstract or keywords of the studies was taken into account during the selection process.

Studies for hypersensitivity, endodontic treatments, dentine pre-treatment and materials without biomodification were excluded. In addition, dissertations, theses or monographs, books, book chapters, letters to the editor, recommendations, systematic or narrative reviews, editorials and errata were also excluded.

### 2.3. Screening and Selection of Articles

**Figure 1:**
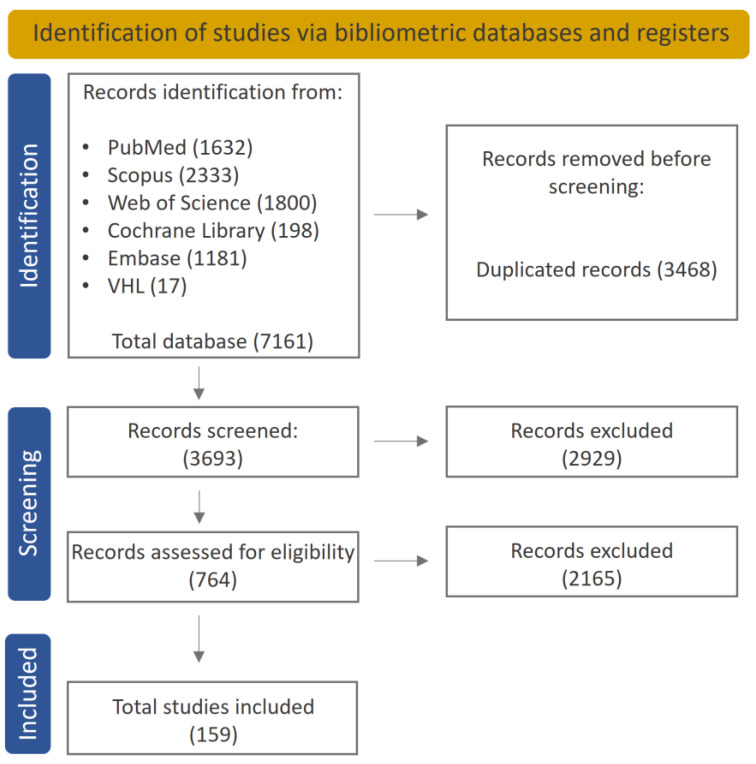
Diagram showing the studies included in the critical review.

All the selected records were imported into text-mining and bibliometric data analysis software (VantagePoint^®^, version 13.0) Initially, an automatic process was used to identify and remove duplicate records. Subsequently, a manual review was conducted to ensure the elimination of any remaining duplicates (Figure 1).

Titles and abstracts were collected by two independent researchers (P.M.P. and T.C.R.), who would check the keywords and the information related to the inclusion criteria before proceeding with the reading in full text. Studies with insufficient data in these sections were read in full, and if the information needed to decide upon eligibility was not available, the study was excluded. Disagreements were resolved by consensus meeting with two other experienced researchers (A.A.N. and L.C.M.), when necessary. After reading in detail, the studies that met all the selection criteria were included to have data extracted and further analyzed.

### 2.4. Data Elements and Collection Method for Study Features

Extracted data from selected articles were manually collected by one researcher (P.M.P.) and checked by another (T.C.R.). In case of any discrepancies, a consensus meeting involving a third researcher (A.A.N.) was held to reach an agreement. Each study was classified according to study design, type of material tested, substrate, analytical method and bioactivity. A single study could fall into multiple categories or subcategories, which is why the final count may not align precisely with the total number of included articles.

In the “study design” category, the subcategories were in vitro, in vivo and ex vivo. For “type of material tested”, the study could be classified according to the biomodified material tested: adhesive, cement or composite. The “substrate” category was subdivided into sound or demineralized dentine. If the dentine was classified as demineralized, the study was then classified according to the demineralization process: chemical, microbiological or natural. For “analytical method”, the subcategories were ion-releasing capability, bond strength, hardness, antibacterial effect, interfacial analysis, chemical characterization and others. Finally, studies were subdivided if they presented bioactivity as yes, no or no difference.

### 2.5. Data Elements and Collection Method for Bibliometric Analysis

The metric analyses encompassed several variables, including “title”, “keywords”, “authors”, “year of publication”, “journal” and “corresponding author’s affiliation country”. The recovery rate was over 90%. The bibliometric assessment of the collected data was conducted using VantagePoint^®^ and Microsoft Excel^®^ (Version 2110).

For author metrics, those with 5 or more studies published were considered, together with the autocorrelation between main authors. Co-occurrences between the main authors and the type of material tested were also analyzed. In addition, a world map illustrating the distribution of studies conducted in each country was generated based on the corresponding authors’ affiliation country data, encompassing all the included studies. Regarding the main published journals, 3 or more studies published were considered and the SCImago Journal Rank (SJR) for each journal was verified using the website https://www.scimagojr.com, with the reference year being 2021. Furthermore, the top 10 most cited articles on this topic were analyzed, including the authors, year and the type of material tested. The most frequently used author keywords were identified from articles and are presented in a word cloud.

The growth of scientific articles published over the years, categorized by the type of material, was visualized using a bubble chart. Additionally, the co-occurrence between the type of material and study design was evaluated, as well as the co-occurrence between the type of material and the substrate, including the type of dentine demineralization process, when available. The analytical methods used and the bioactivity were evaluated due to the co-occurrence between the type of material.

## 3. Results

A total of 7161 records were recovered on the topic of bioactive restorative materials for dentine treatment. After 3468 duplicated records were excluded, 3693 articles remained for a first reading of the title/abstracts/keywords. Subsequently, 605 articles were eliminated for not complying with the eligibility criteria, and thus, 764 articles were included for full-text reading. After this, 159 studies were included. Figure 1 illustrates the flowchart depicting the search selection procedures.

**Figure 2:**
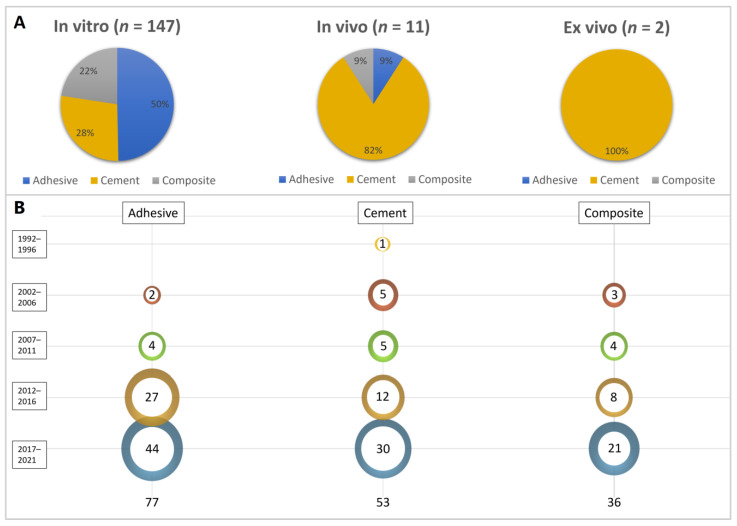
Type of material analysis. (**A**) Distribution of type of material tested according to the study design. (**B**) Scientific publications’ growth by material type over time. The size of each bubble indicates the number of studies published during the analyzed period, with larger bubbles representing a higher number of publications.

For the “study design” category, 149 articles were classified as in vitro, 11 as in vivo and 2 as ex vivo. For the ex vivo subcategory, only one “type of material tested” was evaluated (cement), while for in vitro and in vivo, all types of materials were tested (adhesive, composite and cement). However, for in vitro studies, the most tested materials were adhesives, while for in vivo studies, cements were mostly used (Figure 2A).

**Figure 3:**
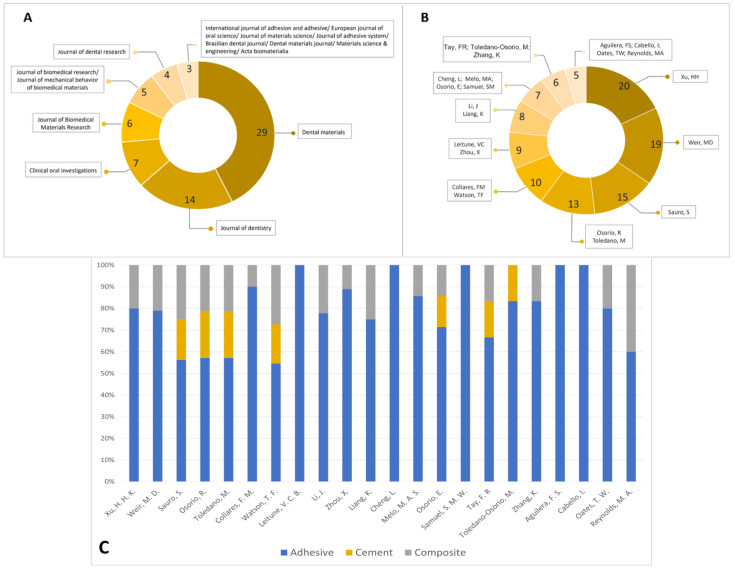
Publication analysis. (**A**) Distribution of publication among the journals. (**B**) Number of publications according to authors. (**C**) Distribution of type of materials tested for each author.

According to the “publication year”, the first article published on the topic of biomodified restorative materials for dentine was in 1994 using a silicate-based cement biomodified with hydroxyapatite. After 2000, there was a rapid increase in the number of publications on the topic, including different types of materials being studied, as shown in Figure 2B. Of the 159 studies identified as eligible, 68 were published in journals where 3 or more articles were published on the topic (Figure 3A). *Dental Materials* was the most published journal (*n* = 29; SJR: 5.304), followed by *Journal of Dentistry* (*n* = 14; SJR: 4.379) and *Clinical Oral Investigations* (*n* = 7; SJR: 3.573).

Regarding the number of publications by authors, a total of 112 articles were detected where one of the listed authors had published more than 5 articles on the topic (Figure 3B). Figure 3C shows the distribution of materials studied among the most prolific authors.

**Figure 4:**
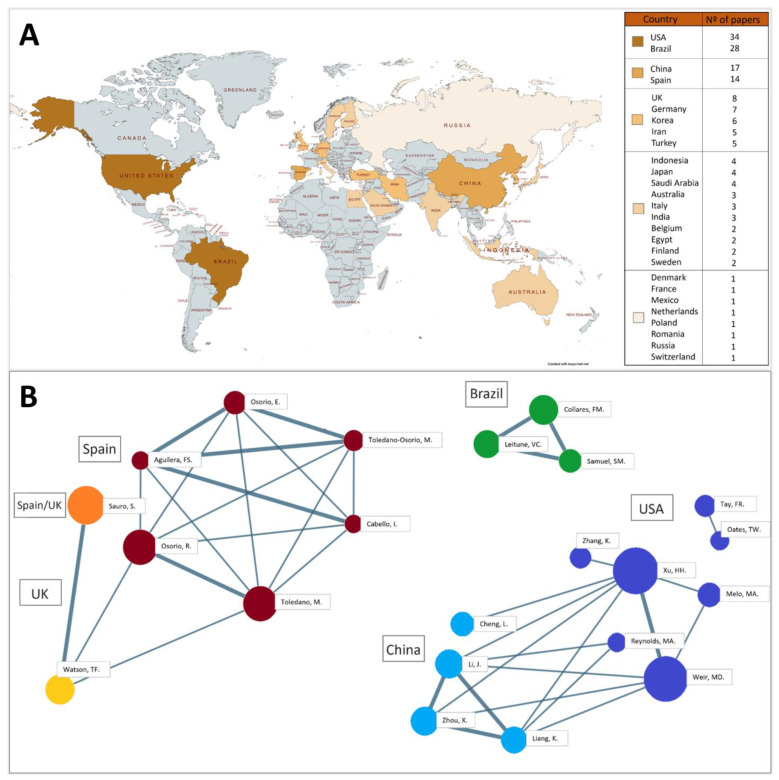
Publication and author analysis. (**A**) World map illustrating the number of studies per country. Colors represent the study count, with gray indicating countries without any publications on the subject. (**B**) Network of authors’ collaboration.

The corresponding author’s affiliation country in each study revealed a total of 27 countries, as depicted in Figure 4A. The USA was the country that had the highest number of published articles (*n* = 34), followed by Brazil (*n* = 28), China (*n* = 17) and Spain (*n* = 14). The collaboration network by authors is shown in Figure 4B, and involved the USA, China, Brazil, Spain and the UK. The ten most cited articles (October 2021) in the Scopus database are shown in Table 2. Half of these were published in *Dental Materials* and the USA was the affiliation country of most of them (*n* = 4).

**Figure 5:**
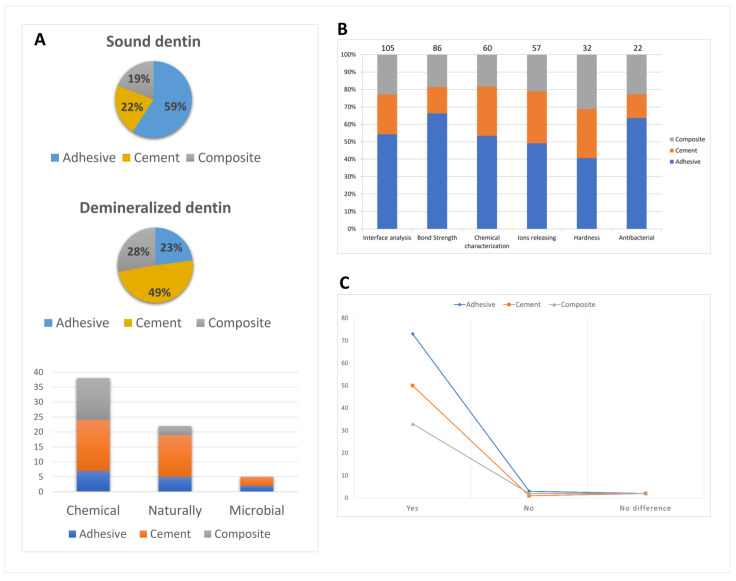
Material analysis. (**A**) Distribution of substrate according to the type of material tested: sound dentine or demineralized dentine and demineralization process according to the type of material tested. (**B**) Distribution of analytical methods used according to the type of material tested for “in vitro” studies. (**C**) Presence, absence or not statistically significant bioactivity in the materials (adhesives, cements and composites) tested by the surveyed studies.

According to the category “substrate studied”, most of the studies were conducted on sound dentine (*n* = 115), adhesives being the most tested material (Figure 5A). For demineralized dentine (*n* = 61), cements were the most tested material. This distribution represents two different approaches for the clinical applications of bioactive materials: on sound dentine, bioactive adhesives are used as sources of remineralization of excessively etched dentine, to avoid leaving exposed collagen inside the hybrid layer. For application in demineralized dentine, the remineralization of residual caries is probably the tested approach. Regarding the demineralization process, chemical models were the first choice (*n* = 38), followed by natural caries (*n* = 22) and microbial models (*n* = 5), cements being the most tested material of all of them.

**Figure 6:**
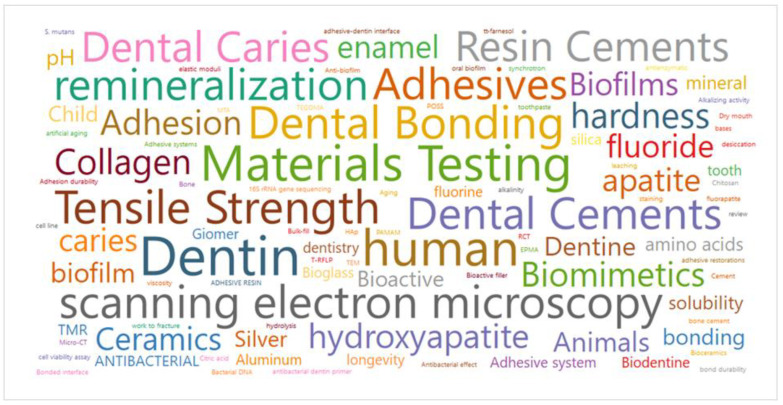
Network visualization of author keywords.

In relation to the “analytical method” subcategory, for “in vivo” and “ex vivo” studies (total of 13 studies), all of them evaluated clinical parameters such as survival of restorations, color match, marginal adaptation, etc. Figure 5B shows the distribution of the analytical methods used for the “in vitro” studies (*n* = 147). It is possible to note that interface analysis was the most investigated (*n* = 105), followed by bond strength (*n* = 86), chemical characterization (*n* = 60), ion release (*n* = 57), hardness (*n* = 32) and antibacterial effect (*n* = 22). The majority of the studies included bioactivity (yes = 148, no = 6, no difference = 5), as shown in Figure 5C. The 100 most cited keywords by the authors are presented in Figure 6, being “dentin” (*n* = 161), “materials testing” (*n* = 97) and “dental bonding” (*n* = 69) the most frequently observed.

## 4. Discussion

The increasing demand for improved dental restorations has driven the ongoing evolution of restorative materials. The preservation of natural tooth tissues and the adoption of minimally invasive approaches have gained significant support, shifting the paradigm in modern dentistry [12]. Consequently, new materials have been developed to meet these demands [13]. The science of biomaterials for restorative dentistry is derived from materials science, but now, interdisciplinary approaches are being adopted by researchers to expand the horizon of biomaterials and maximize their clinical benefits [14]. This is understandable because biomaterials science intersects with other biological sciences. However, it might be responsible for some controversial definitions and classifications, such as “biomimetic”, “bioactive”, “ion-releasing” and “smart materials”, used according to the different interactions between the materials and the tooth substrate [7,15]. There seems to be confusion within the dental profession (among scientists, practitioners and corporate members) regarding to what extent materials can be appropriately termed.

Within these classes, dental materials which are able to leach ions, with a potential role in biomineralization, are glass polyalkenoate and calcium silicate cements [16,17]. In this study, they were classified according to the “type of material” as “cements”. Dental composites are made of relatively biostable thermoset resin matrix and particulate glass or ceramic fillers that are not intended to leach ions or interact in some way with the interface [10]. That is why new formulations of resin composites and adhesive systems are being developed by incorporating specific compounds to improve the interaction with dental tissues [18].

In fact, a significant rise has been observed in the modification of inert materials to induce specific and intentional interactions at the material–tissue interface [1]. The study’s findings indicate a significant increase in the number of publications in this field over the years (Figure 2B). This growth can be attributed to the application of nanotechnology in dentistry and dental tissue engineering [19]. Furthermore, the knowledge of dental caries’ histopathology in accordance with minimal invasive dentistry concepts might contribute to this improvement [20,21].

The shift in the way practitioners manage diseased or damaged hard dental tissues has been very clear. However, most of the current advancements in “smart dental materials” are clearly based on “in vitro” studies (Figure 2A). For in vivo and ex vivo studies, cements were the most tested type of material, probably because interaction with the living tissue is more expected since they are not classified as inert materials, even when small modifications to the formulation are made [14,22]. Sound dentine was the most tested substrate (Figure 5A), where mainly adhesive materials were tested. These materials require a stable bond at the interface, which is not the case when caries-affected dentine is used [23,24,25]. On the other hand, for demineralized dentine, cements were the most tested type of material.

Regarding the demineralization process, chemical models were the first choice, followed by natural caries lesion and microbial models (Figure 5A). An established chemical model for caries lesions can be found in the literature for in vitro studies [26,27]. As most of the studies included in this review were in vitro, this choice can be explained. In these models, dentine caries is simulated by partial demineralization of sound dentine using pH cycling and, in some studies, total demineralization. Microbial models contemplate the etiology of caries lesions, but unfortunately, no specific protocol is well established [28,29,30].

We identified three articles with 100 or more citations in this review (Table 2), which could be then classified as a “classic article” [31]. All of them were published in *Dental Materials*, the journal with the highest number of publications on the topic (Figure 3A) with an impact factor of 5.304, ranking it 8 out of 91 in dentistry, oral surgery and medicine journals. It is known that the number of citations a publication receives could indicate other researchers’ interest in using the information for their own research [32]. The geographic distribution of publications highlights the global interest and collaborative efforts among researchers in understanding and developing new formulations for this type of restorative material (Figure 4A). The study demonstrates the existence of four main clusters of research groups on a global scale: the United States, Brazil, China and Spain (Figure 4B). These can be considered impactful because of the large number of articles published (Figure 4A).

Publications mainly focused on interfacial analysis and bond strength, followed by chemical characterization, ion-releasing ability, hardness recovery and antimicrobial activity (Figure 5B). As bioactivity can be represented by a material that can improve the mechanical properties and bond strength, depending on the dissolution behavior of ions from the surface, these analyses were employed to evaluate the performance of these restorative materials [5,6]. For the cements, the dissolution of calcium aluminofluoro silicate glass particles promotes interaction with the hydroxyl apatite of dentine and the process can be considered bioactivity [7,33]. The significant use of various bioceramics in dentistry is due to their potential for the controlled release of supersaturated ions of calcium and phosphate [14,34]. This may enhance the longevity of restorations, reduce the chance of bacterial growth and increase cell proliferation at the tissue–material interface [3]. To improve the properties of adhesives and resin composites, certain functional additives have been added to the traditional formulations, e.g., bioactive components such as hydroxyapatite and bioactive glasses [35,36]. In fact, one of the limitations of this review was that we did not explore these additives, being a recent trend in dental biomaterials research.

The final category evaluated by this study was related to bioactivity, with almost all studies showing some potential (Figure 5C). However, none of the biomodified restorative materials can be regarded as ideal for clinical indications due to the lack of in vivo studies. This is a large current gap in dental materials research in university laboratories, dental industry and dental practices and this should be overcome in the coming years.

Thus, the overall purpose of this study was to provide a comprehensive bibliometric review of the research published on biomodified restorative materials applied to coronary dentine. Most of the publications with the highest citations were mainly from the United States and published in *Dental Materials*. Nevertheless, the trends indicate a collaborative effort among multiple authors in publishing papers on this topic. However, there is a noticeable lack of clinical studies in this field. This review provides valuable insights for researchers to assess the specific material needs and properties, guiding the design of future research topics. Additionally, it has the potential to facilitate the development of new modified materials and bridge the gap between laboratory research and dental practice. By addressing these aspects, this review aims to foster advancements in the field and promote the translation of research findings into practical dental applications.

## Figures and Tables

**Table 1:** Search strategy according to the different databases used in this study.

Database	Strategy
PubMed	((dentin[MeSH Terms]) OR (dentin*[Title/Abstract])) AND ((((((((((((((((bioactiv*[Title/Abstract]) OR (biomater*[Title/Abstract])) OR (“ion releasing”[Title/Abstract])) OR (“ions releasing”[Title/Abstract])) OR (ion releasing material[Title/Abstract])) OR (ions releasing mater*[Title/Abstract])) OR (“Smart Materials”[Mesh])) OR (“Smart Materials”[Title/Abstract])) OR (“Smart Material”[Title/Abstract])) OR (“Biomimetic Materials”[Mesh])) OR (“biomimetic mater*”[Title/Abstract])) OR (Biomimetics[Mesh])) OR (Biomimetic*[tiab])) OR (“Smart Dentin Replacement”[Supplementary Concept])) OR (“Smart Dentin Replacement”[tiab])) OR (Dentin* Replacement[Title/Abstract]))
Scopus	(TITLE-ABS-KEY (dentin) OR TITLE-ABS-KEY (dentin*) AND TITLE-ABS-KEY (bioactiv*) OR TITLE-ABS-KEY (biomater*) OR TITLE-ABS-KEY (“ion releasing”) OR TITLE-ABS-KEY (“ions releasing”) OR TITLE-ABS-KEY (“ion releasing material”) OR TITLE-ABS-KEY (ions AND releasing AND mater*) OR TITLE-ABS-KEY (“smart materials”) OR TITLE-ABS-KEY (“smart material”) OR TITLE-ABS-KEY (“biomimetic materials”) OR TITLE-ABS-KEY (biomimetic AND mater*) OR TITLE-ABS-KEY (biomimetics) OR TITLE-ABS-KEY (biomimetic*) OR TITLE-ABS-KEY (“smart dentin replacement”) OR TITLE-ABS-KEY (dentin*replacement))
Web of Science	TS = (dentin) OR TS = (dentin*) AND TS = (bioactiv*) OR TS = (biomater*) OR TS = (“ion relasing”) OR TS = (“ions releasing”) OR TS = (“ion releasing material”) OR TS = (ions releasing mater*) OR TS = (“smart materials”) OR TS = (“smart material”) OR TS = (“biomimetic materials”) OR TS = (biomimetic mater*) OR TS = (biomimetics) OR TS = (biomimetic*) OR TS = (“smart dentin replacement”) OR TS = (dentin*replacement)
Cochrane Library	ID Search Hits 1 MeSH descriptor: [Dentin] explode all trees 1260 2 (dentin*):ti,ab,kw 4468 3 (bioactiv*):ti,ab,kw 2166 4 (biomater*):ti,ab,kw 573 5 (“ion releasing”):ti,ab,kw 10 6 (“ions releasing”):ti,ab,kw 0 7 (“ion releasing material”):ti,ab,kw 2 8 (ions releasing mater*):ti,ab,kw 5 9 MeSH descriptor: [Smart Materials] explode all trees 1 10 (“Smart Material”):ti,ab,kw 1 11 MeSH descriptor: [Biomimetic Materials] explode all trees 1340 12 (biomimetic mater*):ti,ab,kw 54 13 MeSH descriptor: [Biomimetics] explode all trees 11 14 (Biomimetic*):ti,ab,kw 124 15 (“Smart Dentin Replacement”):ti,ab,kw 7 16 (Dentin* Replacement):ti,ab,kw 71 17 1 OR 2 4468 18 3 OR 4 OR 5 OR 6 OR 7 OR 8 OR 9 OR 10 OR 11 OR 12 OR 13 OR 14 OR 15 OR 16 4208 19 17 AND #8 198
VHL (BBO/LILACS)	(mh: dentin OR dentin*) AND (bioactive* OR biomater* OR “ion releasing” OR “ions releasing” OR “ion releasing material” OR ions releasing mater* OR mh: “smart materials” OR “smart material” OR mh: “biomimetic materials” OR biomimetric mater* OR mh: biomimetics OR biomimetic* OR “smart dentin replacement” OR dentin*replacement) AND (db:(“LILACS”))
EMBASE	dentin:ti,ab,kw OR dentin*:ti,ab,kw AND bioactiv*:ti,ab,kw OR biomaterial:ti,ab,kw OR biomater*:ti,ab,kw OR ‘ion releasing’:ti,ab,kw OR ‘ions releasing’:ti,ab,kw OR ‘ion releasing material’:ti,ab,kw OR ‘ions releasing mater*’:ti,ab,kw OR ‘smart materials’:ti,ab,kw OR ‘smart material’:ti,ab,kw OR ‘biomimetic materials’:ti,ab,kw OR ‘biomimetic material’:ti,ab,kw OR biomimetics:ti,ab,kw OR biomimetic*:ti,ab,kw OR ‘smart dentin replacement’:ti,ab,kw OR ‘dentin* replacement’:ti,ab,kw

* Means search includes the root of a word to find multiple endings.

**Table 2:** Ten most cited articles published, their affiliation country, first author, publication year, journal, type of material tested, and times cited.

Study	Country	First Author	Year	Journal	Material	Times Cited
Hydroxyapatite nanorods as novel fillers for improving the properties of dental adhesives: Synthesis and application	Iran	Sadat-Shojai, M	2010	Dent Mater	Adhesive	181
Mechanical properties and biochemical activity of remineralizing resin-based Ca-PO4 cements	USA	Dickens, S.H	2003	Dent Mater	Cement	155
Novel dental adhesives containing nanoparticles of silver and amorphous calcium phosphate	USA	Melo, MA	2013	Dent Mater	Adhesive	132
Toughness, bonding and fluoride-release properties of hydroxyapatite-added glass ionomer cement	Japan	Lucas, ME	2003	Biomater	Cement	99
Novel dental adhesive containing antibacterial agents and calcium phosphate nanoparticles	USA	Melo, MA	2013	J Biomed Mater Res B Appl Biomater	Adhesive	90
Therapeutic effects of novel resin bonding systems containing bioactive glasses on mineral-depleted areas within the bonded-dentine interface	UK	Sauro, S	2012	J Mater Sci Mater Med	Composite	90
Biomimetic remineralization of human dentin using promising innovative calcium-silicate hybrid “smart” materials	Italy	Gandolfi, MG	2011	Dent Mater	Composite	84
Anti-biofilm dentin primer with quaternary ammonium and silver nanoparticles	USA	Cheng, L	2012	J Dent Res	Adhesive	80
Antibacterial activity and ion release of bonding agent containing amorphous calcium phosphate nanoparticles	China	Chen, C	2014	Dent Mater	Adhesive	76
Remineralization of artificial dentinal caries lesions by biomimetically modified mineral trioxide aggregate	China	Qi, YP	2012	Acta Biomater	Cement	75

## Data Availability

The data presented in this study are available on request from the corresponding author. The data are not publicly available due to university privacy.
